# Purification and Characterization of JZTx-14, a Potent Antagonist of Mammalian and Prokaryotic Voltage-Gated Sodium Channels

**DOI:** 10.3390/toxins10100408

**Published:** 2018-10-10

**Authors:** Jie Zhang, Dongfang Tang, Shuangyu Liu, Haoliang Hu, Songping Liang, Cheng Tang, Zhonghua Liu

**Affiliations:** The National and Local Joint Engineering Laboratory of Animal Peptide Drug Development, College of Life Sciences, Hunan Normal University, Changsha 410081, China; jiezhang@smail.hunnu.edu.cn (J.Z.); tangdf@smail.hunnu.edu.cn (D.T.); liushuangyu@smail.hunnu.edu.cn (S.L.); haolianghu6@126.com (H.H.); liangsp@hunnu.edu.cn (S.L.)

**Keywords:** NaChBac, mammalian Na_V_s, peptide toxin, pharmacology

## Abstract

Exploring the interaction of ligands with voltage-gated sodium channels (Na_V_s) has advanced our understanding of their pharmacology. Herein, we report the purification and characterization of a novel non-selective mammalian and bacterial Na_V_s toxin, JZTx-14, from the venom of the spider *Chilobrachys jingzhao*. This toxin potently inhibited the peak currents of mammalian Na_V_1.2–1.8 channels and the bacterial NaChBac channel with low IC_50_ values (<1 µM), and it mainly inhibited the fast inactivation of the Na_V_1.9 channel. Analysis of Na_V_1.5/Na_V_1.9 chimeric channel showed that the Na_V_1.5 domain II S3–4 loop is involved in toxin association. Kinetics data obtained from studying toxin–Na_V_1.2 channel interaction showed that JZTx-14 was a gating modifier that possibly trapped the channel in resting state; however, it differed from site 4 toxin HNTx-III by irreversibly blocking Na_V_ currents and showing state-independent binding with the channel. JZTx-14 might stably bind to a conserved toxin pocket deep within the Na_V_1.2–1.8 domain II voltage sensor regardless of channel conformation change, and its effect on Na_V_s requires the toxin to trap the S3–4 loop in its resting state. For the NaChBac channel, JZTx-14 positively shifted its conductance-voltage (G–V) and steady-state inactivation relationships. An alanine scan analysis of the NaChBac S3–4 loop revealed that the 108th phenylalanine (F108) was the key residue determining the JZTx-14–NaChBac interaction. In summary, this study provided JZTx-14 with potent but promiscuous inhibitory activity on both the ancestor bacterial Na_V_s and the highly evolved descendant mammalian Na_V_s, and it is a useful probe to understand the pharmacology of Na_V_s.

## 1. Introduction

The mammalian voltage-gated sodium channel (Na_V_) is composed of a pore-forming alpha subunit and one or two covalently or non-covalently associated beta subunits. The alpha subunit has a topological structure of 24 transmembrane segments (TMs), which could be further divided into four homologous domains, with each domain containing six TMs. A total of nine Na_V_ subtypes have been identified in mammals with different tissue distributions and accordingly diverse functions: the Na_V_1.1–1.3 subtypes that are mainly located in the central nervous system (CNS); the Na_V_1.4–1.5 subtypes that are expressed in the skeletal muscle cells and the cardiac myocyte cells, respectively; the Na_V_1.7–1.9 subtypes that are mostly restricted to the peripheral nervous system (PNS); and the Na_V_1.6 channel, which is expressed in both the CNS and the PNS [1,2]. These subtypes construct the molecular bases of the excitability in these cells, and their functional dysregulation is closely related to diseases such as pain, epilepsy, ataxia, Brugada/QT syndrome, etc. [3]. Furthermore, recent studies have shown that Na_V_s were expressed in non-excitable cells such as astrocytes, microglia, macrophages, and cancer cells, and functioned in a non-canonical way [4]. The gating and the Na^+^ selectivity mechanism of mammalian Na_V_s are yet to be explored, and the milestone progresses regarding the cryo-EM structures of eukaryotic Na_V_s such as cockroach Na_V_Pas [5], eel EeNa_V_1.4 [6], and human Na_V_1.4 [7] have shed light on this issue. The venoms of toxic animals are rich in toxins modulating the activity of Na_V_s, and are rich mines for developing drugs for treating Na_V_-related diseases and discovering probes for Na_V_ researches [8,9]. To date, eight neurotoxin sites have been characterized in mammalian Na_V_s; among these, site 1, site 3, site 4, and site 6 are binding sites for small disulfide-rich peptides from animal venoms [10]. These peptide toxins function by either physically occluding the pore or affecting the voltage sensor movement driven by membrane potential change [11]. Given the high homology between Na_V_1.2–1.8 channels, it is difficult to purify Na_V_ subtype-specific modulators from animal venoms. This is because that these toxins might bind to Na_V_s by recognizing a common structure in them, which is similar to that of tetrodotoxin (TTX) occluding the pore of TTX-sensitive Na_V_s (Na_V_1.1–1.4, Na_V_1.6–1.7) by recognizing the conserved structures in them [12,13]. However, peptide toxins that act on all of the nine Na_V_ subtypes with comparable affinity are rare, as there are substantial differences between them. Furthermore, Na_V_ voltage sensor binding peptide toxins (site 3 and site 4 toxins) have always shown reversible and state-dependent binding property. The identification of toxins deviating from these rules would advance our understanding of the pharmacology of Na_V_ channels.

Mammalian Na_V_s have their structural and functional relatives in prokaryotic organisms, namely the bacterial Na_V_s. The first bacterial Na_V_, NaChBac, was characterized in *Bacillus halodurans*, and successfully expressed Na^+^ selective current when transfected into the CHO-K1 cell line [14]. Since then, numerous bacterial Na_V_s have been discovered in different bacteria species, and all of them have a quaternary structure of four subunits assembling into the functional channel, which is similar to that of the voltage-gated potassium channel. Each bacterial Na_V_ subunit has six transmembrane segments, which are analogous to one homologous domain in mammalian Na_V_s [15,16]. This simplified topological structure has facilitated the use of bacterial Na_V_s in structural analysis via a crystallography strategy. Recently, several bacterial Na_V_ structures with atomic resolution have been reported [17,18,19,20,21,22,23], which provided valuable opportunity to explore the pathogenic mechanism, the gating, the ion selectivity, and the pharmacology of mammalian Na_V_s from a structural perspective, as they have similar functional components. For instance, the crystal structure of the bacterial NavAb channel with gating charges mutation revealed the pathogenic mechanism of periodic paralysis underlain by human Na_V_1.4 mutation at the atomic level [24]. The crystal structures of the NavAb channel with the activation gate captured in the close and open conformations uncovered the gate’s C–O–I conformational cycle, which provided important information for understanding the activation gating in eukaryotic Na_V_s [25]. Furthermore, modeling mammalian Na_V_ pores based on the prokaryotic Na_V_Rh structure revealed the role of lysine at the constriction site in Na^+^ selectivity, and the possibility of a common underlying foundation of the selective conduction of Na^+^ in them [26,27]. In the pharmacological study of bacterial Na_V_s, bacterial and mammalian Na_V_s were found to share their sensitivity to drugs targeting their inactivation, such as lidocaine and isoflurane [19,28]. Additionally, it was proposed that the bacterial Na_V_M channel structure can be used as a three-dimensional (3D) template for designing drugs targeting human Na_V_s [19]. However, compared with mammalian Na_V_s, relatively few peptide toxins acting on bacterial Na_V_s have been reported. 

In the present study, a novel peptide toxin, JZTx-14, from the venom of the spider *Chilobrachys jingzhao*, is purified and characterized. It showed promiscuous inhibitory activity on mammalian Na_V_1.2–1.8 channels and the bacterial NaChBac channel, and also inhibited the fast inactivation of the Na_V_1.9 channel. Kinetics data and mutation analysis proved that JZTx-14 was a gating modifier of both mammalian and bacterial Na_V_s. However, its irreversible blocking effect on mammalian Na_V_ and its state-independent binding with the channel suggested that the action mode of JZTx-14 might be different from those classical site 4 toxins: that is, JZTx-14 might stably bind to a conserved toxin pocket deep within the Na_V_1.2–1.8 voltage sensor regardless of channel conformation change, however, its inhibitory effect on Na_V_s requires the toxin to trap the S3–4 loop in its resting state. This study provided a novel toxin to understand the pharmacology of both the mammalian and bacterial Na_V_s.

## 2. Results

### 2.1. Purification and Characterization of JZTx-14

**Figure 1:**
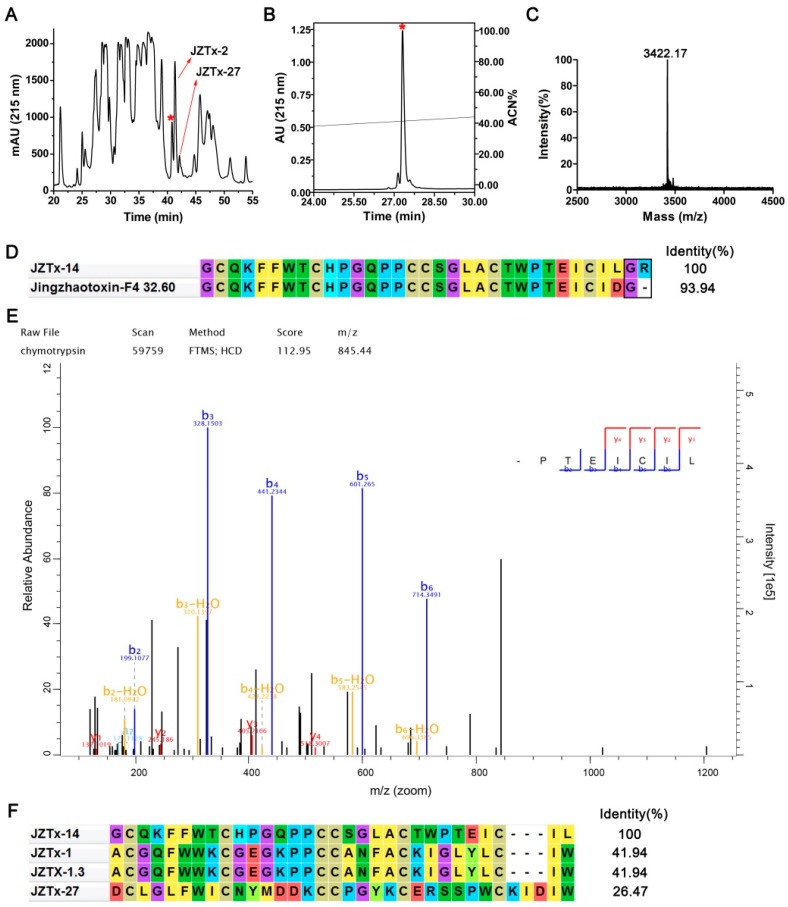
Purification and characterization of JZTx-14. (**A**) RP-HPLC purification profile of *Chilobrachys jingzhao* venom. The peaks containing JZTx-14, JZTx-2, and JZTx-27 are labeled by asterisk and arrows, respectively. (**B**) JZTx-14 was purified to homogeneity by analytical RP-HPLC. (**C**) MALDI-TOF MS analysis of purified JZTx-14. The average molecular mass of JZTx-14 was determined as 3422.17 Da (M + H^+^). (**D**) The mature peptide sequences of JZTx-14 and Jingzhaotoxin F4-32.60. The C-terminal amidation signals G in Jingzhaotoxin F4-32.60 and GR in JZTx-14 were boxed. (**E**) Chymotrypsin digestion combined with LC-MS analysis determined the toxin to be JZTx-14. The MS/MS spectrum of the C-terminal fragment is shown (sequence: TEICIL). (**F**) Sequence alignment of JZTx-14 with similar toxins in database. The bacterial Na_V_ toxin JZTx-27 is included.

In the present study, we purified and characterized a toxin from the venom of the spider *C. jingzhao*, JZTx-14, which non-selectively and potently inhibited Na_V_1.2–1.8 currents. This toxin had a retention time of 40.8 min in the RP-HPLC purification of the venom (Figure 1A, red asterisk indicated peak). It was also active on bacterial Na_V_s NaChBac and NsvBa, as revealed by our previous screening analysis of spider venom peptides for bacterial Na_V_s antagonists. However, the toxin was not deeply explored as a bacterial Na_V_ toxin, as it was approximately 10 folds less potent than the toxin JZTx-27, which was investigated in our previous study [29]. This toxin was purified to homogeneity (Figure 1B,C), and its molecular weight was determined as 3422.17 Da (M + H^+^) (Figure 1C). Additionally, we determined its N-terminal sequence of 11 amino acids by Edman degradation (GCQKFFWTCHP), and blasting this sequence in the non-redundant protein sequences database by using the NCBI blast tool (https://blast.ncbi.nlm.nih.gov/Blast.cgi) matched the toxins JZTx-14 and Jingzhaotoxin F4-32.60 (Figure 1D). Jingzhaotoxin F4-32.60 differed from JZTx-14 by the C-terminal residue (Aspartate in Jinzhaotoxin F4-32.60 and Leucine in JZTx-14; note the residues GR in JZTx-14 and the residue G in Jingzhaotoxin F4-32.60 were amidation signals, Figure 1D). We analyzed the C-terminal sequence of the toxin by combining chymotrypsin digestion and LC–MS analysis. Searching the LC–MS data against the venom gland cDNA library database of *Chilobrachys jingzhao* matched JZTx-14 with the highest score (UniProtKB/Swiss-Prot accession number: B1P1C0.1), with an identification coverage ratio of 100%. Figure 1E shows the C-terminal peptide MS/MS spectrum of JZTx-14. The theoretical molecular weight of JZTx-14 (3428.05 Da) was 7 Da more than that determined experimentally, which suggested the six cysteines in JZTx-14 formed three disulfide bonds and the C-terminus amidation. The cysteine framework in JZTx-14 is conserved in most of the ICK motif spider toxins (C_1_-C_2_-C_3_C_4_-C_5_-C_6_), and the mode of the disulfide bonds might be C_1_-C_4_, C_2_-C_5_, and C_3_-C_6_ (the number indicates the relative position of cysteine in the sequence). Sequence alignment showed that JZTx-14 had low identity with the peptide toxins in the database (Figure 1F).

### 2.2. JZTx-14 Is a Potent but Non-Selective Mammalian Na_V_s Toxin

**Figure 2:**
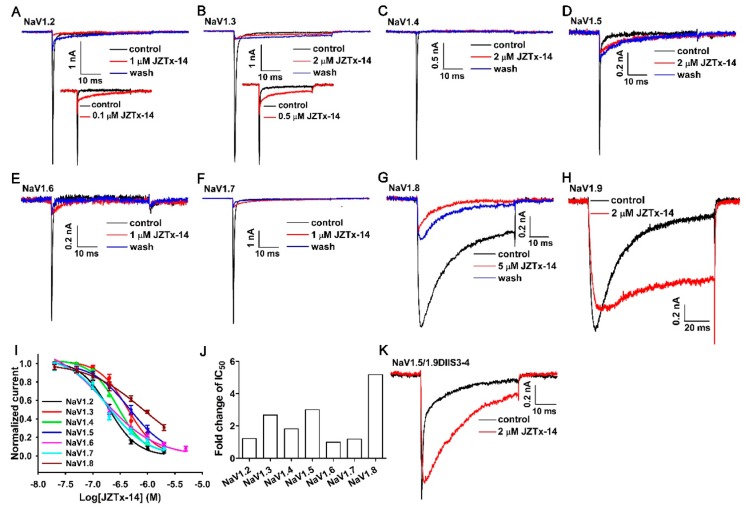
Activity of JZTx-14 on mammalian Na_V_s. (**A**–**H**) Representative current traces showing that JZTx-14 blocked the currents of Na_V_1.2–1.8 and slowed the fast inactivation of Na_V_1.9 (black traces: control; red traces: after toxin application; blue traces: after 2- to 3-min bath solution perfusion). The insets in (**A**) and (**B**) show that the toxin slowed the fast inactivation of Na_V_1.2 and Na_V_1.3. (**I**) Dose–response curves for JZTx-14 inhibiting Na_V_1.2–1.8 currents. The IC_50_ values were 194.0 ± 10.3 nM, 426.3 ± 48.8 nM, 290.1 ± 23.2 nM, 478.0 ± 32.0 nM, 158.6 ± 29.4 nM, 188.9 ± 46.3 nM, and 824.0 ± 68.7 nM for Na_V_1.2, Na_V_1.3, Na_V_1.4, Na_V_1.5, Na_V_1.6, Na_V_1.7, and Na_V_1.8, respectively (*n =* 4–6). (**J**) The comparison of JZTx-14 affinities to Na_V_1.2–1.8 channels. The IC_50_ value of JZTx-14 for each Na_V_ subtype was normalized to that of the Na_V_1.6 channel. (**K**) The effect of 2 µM JZTx-14 on the Na_V_1.5/1.9DIIS3–4 chimeric channel constructed by substituting the Na_V_1.5 domain II S3–4 loop with that of Na_V_1.9 (*n =* 4).

We tested the activity of JZTx-14 on mammalian Na_V_s Na_V_1.2–1.9 heterologously expressed in HEK293T or ND7/23 cells. The Na_V_1.8 and Na_V_1.9 channels were chimeras, as described in our previous studies [30,31]. Their currents were elicited by 50-ms depolarizations to 0 mV from the holding potential of −80 mV. 1 µM or 2 µM JZTx-14 almost fully blocked Na_V_1.2–1.7 currents (Figure 2A–F, red traces). Low-dose toxin also slightly inhibited the fast inactivation of Na_V_1.2–1.7, with the Na_V_1.2 and Na_V_1.3 channels being the most affected (Figure 2A,B, inset). The Na_V_1.8 channel was relatively less sensitive to JZTx-14 when compared with Na_V_1.2–1.7 channels, with 5 µM toxin inhibiting its currents by approximately 80% (Figure 2G, red trace). Notably, the inhibitory effects of JZTx-14 on Na_V_1.2–1.8 channels were irreversible, as a 2- to 3-min bath solution perfusion could not wash off the toxin (Figure 2A–G, blue traces). For the Na_V_1.9 channel, the toxin potently slowed its fast inactivation and only slightly inhibited its peak current (Figure 2H). The dose–response curves for JZTx-14 blocking the peak currents of these Na_V_ subtypes show that Na_V_1.6 was the most sensitive to toxin, and the IC_50_ values were 194.0 ± 10.3 nM for Na_V_1.2 (*n* = 5), 426.3 ± 48.8 nM for Na_V_1.3 (*n* = 5), 290.1 ± 23.2 nM for Na_V_1.4 (*n* = 6), 478.0 ± 32.0 nM for Na_V_1.5 (*n* = 5), 158.6 ± 29.4 nM for Na_V_1.6 (*n* = 4), 188.9 ± 46.3 nM for Na_V_1.7 (*n* = 6), and 824.0 ± 68.7 nM for Na_V_1.8 (*n* = 5) (Figure 2I,J). These data proved that JZTx-14 was a non-selective antagonist of mammalian Na_V_s. Additionally, the high-affinity binding of JZTx-14 to Na_V_1.2–1.8 subtypes suggests the common molecular determinants in these channels for toxin association. We constructed the Na_V_1.5/1.9DIIS3–4 chimeric channel by substituting the Na_V_1.5 domain II(DII) S3–4 loop with that of Na_V_1.9, and tested its response to JZTx-14. The data showed that 2 µM toxin only slightly inhibited its peak currents, but profoundly slowed its fast inactivation (Figure 2K). This supports a direct interaction of the toxin with the channel, and that the Na_V_1.5 DII S3–4 loop participated in toxin association. We further mutated several amino acids in the Na_V_1.5 DII S3–4 loop to that of Na_V_1.9 (Na_V_1.5/L798V, Na_V_1.5/S799L, Na_V_1.5/S802R, Na_V_1.5/L804W); all of these mutants showed no significant change of their sensitivity to JZTx-14, and an alanine scan mutation analysis of the DII S3b–S4 paddle motif (from the 795th glutamine to the 808th arginine) did not identify any key site (data not shown). We propose that JZTx-14 associated with the DII S3–4 loops of Na_V_1.2–1.8 by binding multiple amino acids in this region. It well explained the targets proximity of JZTx-14 among Na_V_ subtypes.

### 2.3. JZTx-14 Acts on Mammalian Na_V_s as a Gating Modifier

**Figure 3:**
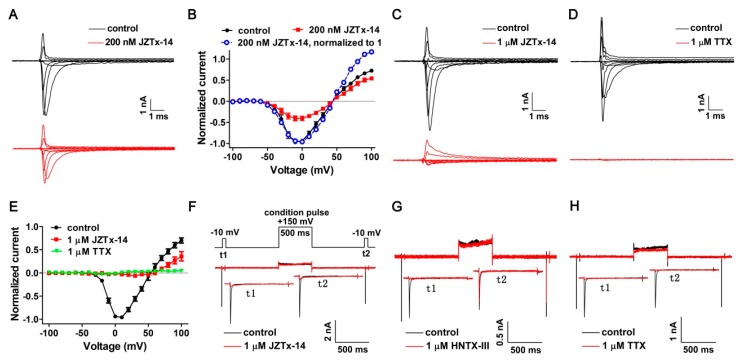
Kinetics of JZTx-14 interacting with Na_V_1.2. (**A**) Representative Na_V_1.2 current traces before and after a subsaturating concentration (200 nM) of JZTx-14 treatment. Currents were elicited by a cluster of depolarizations from −100 mV to +100 mV (in 10-mV increments) from the holding potential of −100 mV. For simplicity, currents in 20-mV increments were shown. (**B**) I–V relationships of the Na_V_1.2 channel before and after 200 nM JZTx-14 treatment (black and red solid lines). Currents after toxin treatment were normalized to 1 (blue dashed line) to compare the I–V shape with that before toxin application (*n =* 10). (**C**,**D**) Representative Na_V_1.2 current traces before and after saturating concentration (1 µM) of JZTx-14 or TTX treatment. Currents were elicited as described in Figure 3A. (**E**) I–V relationship of the Na_V_1.2 channel before and after 1 µM JZTx-14 or TTX treatment. TTX and JZTx-14 almost fully inhibited Na_V_1.2 inward currents, and TTX, but not JZTx-14, fully blocked Na_V_1.2 outward currents (*n =* 5). (**F**–**H**) The protocol in Figure 3F was used to measure toxin dissociation from Na_V_1.2 in response to a +150 mV/500 ms strong depolarization by testing the currents in test pulse 2 (t2), and 1 µM JZTx-14, HNTX-III, or TTX were used to fully block Na_V_1.2 currents in test pulse 1 (t1). A large current recovery was observed in t2 in the HNTX-III group, but not in the JZTx-14 or TTX groups (*n =* 3–5).

Na_V_1.2 was used as a representative channel and the effect of JZTx-14 on its activation was tested. As shown by the traces in Figure 3A, 0.2 µM JZTx-14 inhibited Na_V_1.2 currents at every depolarizing voltage, and the toxin did not change its peak current voltage or reversal voltage (Figure 3B, black and red solid lines; *n* = 10). Additionally, we compared the I–V relationships by normalizing the currents in the toxin group to their maximum peak current (normalized to 1). The data showed that the curves superimposed between the depolarizing voltages of −50 mV to +50 mV, while the proportion of opening channels at voltages of +60 mV to +100 mV in the toxin group was higher than that in the control group (Figure 3B, black solid and blue dashed lines). This suggests that a population of toxin-bound channels were reopened at stronger depolarizations, a supposition that was further validated by testing the I–V relationship of the Na_V_1.2 channel in the presence of a saturating dose of JZTx-14. As shown by the representative traces in Figure 3C, 1 µM JZTx-14 almost fully blocked the Na_V_1.2 inward currents, while the outward currents were partially inhibited. In contrast, the Na_V_ out pore blocker TTX fully inhibited both the Na_V_1.2 inward and outward currents (Figure 3D). These data and the I–V curves shown in Figure 3E suggest a voltage dependent inhibition of JZTx-14, but not TTX, on the Na_V_1.2 channel (*n* = 5). We propose that JZTx-14 trapped the Na_V_1.2 DII voltage sensor in one of the deactivated states. We further conducted the toxin dissociation assay to explore the state-dependent binding of JZTx-14 with Na_V_1.2. As shown by the voltage protocol in Figure 3F, a 500-ms condition pulse (cp) to +150 mV was used to drive voltage sensor outward and subsequent toxin dissociation. Additionally, the test pulse 1 (t1) and test pulse 2 (t2) to −10 mV were used to measure the currents before and after the condition pulse, and the recovery duration at −120 mV between t1 and cp as well as cp and t2 was 800 ms. The tarantula toxin HNTx-III inhibited the Na_V_1.2 currents by binding to the DII voltage sensor. Saturating doses of JZTx-14, HNTx-III, and TTX fully blocked the Na_V_1.2 currents elicited by t1. However, in contrast to a large recovery of the Na_V_1.2 current in t2 in the HNTx-III group, no current recovery was observed in the JZTx-14 and TTX groups (Figure 3F–H; *n* = 3–5). It is reasonable that strong depolarization could not cause TTX dissociation from Na_V_1.2 as its action mechanism. The action mode of JZTx-14, which is characterized by its state-independent binding with Na_V_1.2 and the irreversible blocking of Na_V_1.2 currents, is distinct from other toxins acting on the Na_V_ DII voltage sensor, such as HNTx-III and HWTx-IV [32,33].

### 2.4. Effects of JZTx-14 on Bacterial Na_V_s

**Figure 4:**
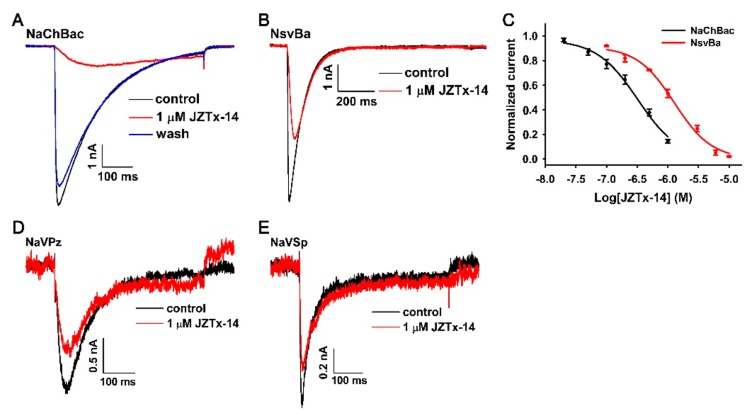
Activity of JZTx-14 on bacterial Na_V_s. (**A**,**B**) Representative traces showing the inhibitory effect of JZTx-14 on the NaChBac and NsvBa channels. (**C**) Dose–response curves for JZTx-14 inhibiting the NaChBac and NsvBa currents. The IC_50_ values were 320 ± 38 nM and 1400 ± 200 nM for NaChBac and NsvBa, respectively (*n =* 5–7). (**D**,**E**) The inhibitory effect of 1 μM JZTx-14 on NaVPz and NaVSp currents (*n =* 3).

**Figure 5:**
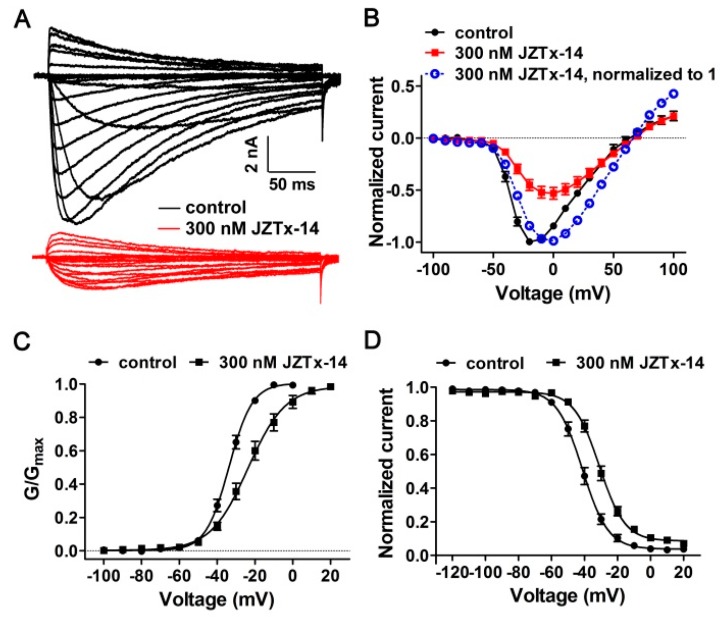
Kinetics of JZTx-14 interacting with NaChBac. (**A**) Representative traces showing that 300 nM JZTx-14 inhibited NaChBac currents at all of the voltages tested. Currents were elicited by depolarizations from −100 mV to +100 mV from the holding potential of −100 mV (in 10-mV increments). (**B**) I–V relationships of NaChBac before and after 300 nM JZTx-14 treatment. Currents were normalized to that before toxin application (red solid traces). The blue dashed line shows the normalization of the currents after toxin treatment to 1 (*n =* 5). (**C**,**D**) Steady-state activation (G–V) and steady-state inactivation (SSI) relationships of NaChBac before and after 300 nM JZTx-14 treatment (V_a_ = −33.8 ± 0.41 mV and −23.7 ± 0.98 mV, K_a_ = 6.2 ± 0.35 mV and 9.9 ± 0.87 mV, for control and toxin treated channels, respectively, *n =* 5; V_h_ = –41.3 ± 0.55 mV and –30.9 ± 0.56 mV, K_h_ = −7.8 ± 0.48 mV and −7.7 ± 0.48 mV, for control and toxin treated channels, respectively; *n =* 5). The curves were fitted by Equation (1).

As shown in Figure 4A, 1 µM JZTx-14 almost fully inhibited the NaChBac channel currents (red trace), and this effect was reversible as the toxin could be washed off by bath solution perfusion (blue trace). However, the potency of JZTx-14 to another bacterial Na_V_, NsvBa, was relatively weaker, with 1 µM toxin inhibiting its currents by 46.8 ± 3.3% (Figure 4B, *n* = 6). The dose–response curves showed that the IC_50_ values of JZTx-14 for the NaChBac and NsvBa channels were 320 ± 38 nM and 1400 ± 200 nM, respectively (Figure 4C, *n* = 5–7). For the bacterial Na_V_s NaVPz and NaVSP, 1 µM JZTx-14 only caused a 34.5 ± 6.5% and 25.7 ± 5.7% inhibition of their currents, respectively (Figure 4D,E, *n* = 3). JZTx-27 is another NaChBac and NsvBa channel antagonist characterized in the same venom [29]. In the RP-HPLC purification of the venom, the JZTx-27 peak followed those of JZTx-14 and JZTx-2 (Figure 1A; the arrows labeled peaks). Interestingly, JZTx-14 and JZTx-27 all showed preference to the NaChBac channel among the tested bacterial Na_V_s. However, compared with JZTx-27, JZTx-14 showed more potent activity on mammalian Na_V_s. As for mammalian Na_V_s, we tested the effect of JZTx-14 on the steady-state activation and inactivation of NaChBac. As shown in Figure 5A, 300 nM toxin inhibited the NaChBac currents at all of the voltages tested. The curve in Figure 5B shows that the toxin positively shifted the I–V relationship of NaChBac without affecting the reversal voltage, indicating that the toxin did not change the ion selectivity of the channel. Additionally, the G–V curve was consistently shifted to the depolarization direction (V_a_ = −33.8 ± 0.41 mV and –23.7 ± 0.98 mV, K_a_ = 6.2 ± 0.35 mV and 9.9 ± 0.87 mV, for control and toxin-treated channels, respectively; Figure 5C, *n* = 5). Furthermore, the toxin positively shifted the steady-state inactivation relationship of NaChBac (V_h_ = −41.3 ± 0.55 mV and −30.9 ± 0.56 mV, K_h_ = −7.8 ± 0.48 mV and −7.7 ± 0.48 mV, for control and toxin-treated channels, respectively; Figure 5D, *n* = 5). These data suggest that JZTx-14 acted on NaChBac as a gating modifier.

### 2.5. The Molecular Mechanism of JZTx-14 Interacting with NaChBac

**Figure 6:**
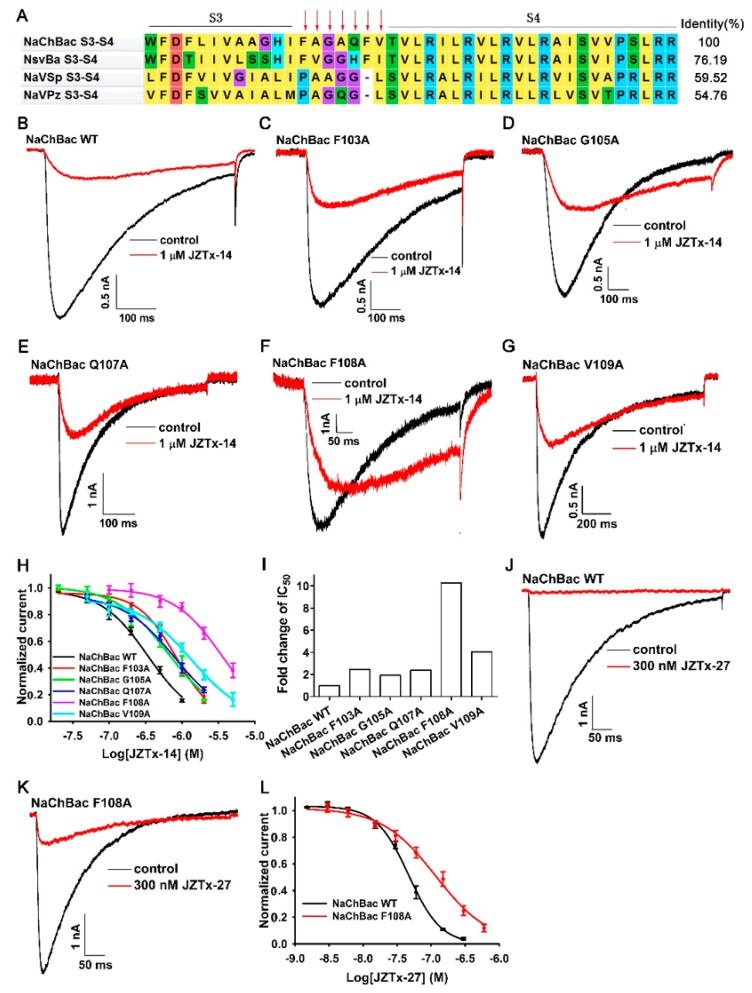
Effect of JZTx-14 on NaChBac mutants. (**A**) Sequence alignment of NaChBac with several bacterial Na_V_s determined the S3–4 extracellular loops. The mutation sites are labeled by red arrows. (**B**–**G**) Representative traces showing the inhibitory effect of 1 µM JZTx-14 on wild-type NaChBac and NaChBac mutants. (**H**) Dose–response curves for JZTx-14 inhibiting the currents of NaChBac mutants. The IC_50_ values were 320.0 ± 38.0 nM, 832.6 ± 42.1 nM, 653.3 ± 92.1 nM, 809.8 ± 87.5 nM, 3472.3 ± 195.7 nM, and 1367.3 ± 129.3 nM for wild-type NaChBac, F103A, G105A, Q107A, F108A, and V109A, respectively (*n =* 5–7). (**I**) The bars show the fold changes of the IC_50_ values of mutant channels when compared with wild-type NaChBac. (**J**,**K**) Representative traces showing the inhibitory effect of 300 nM JZTx-27 on wild-type NaChBac and F108A mutant channel. (**L**) Dose–response curves for JZTx-27 inhibiting the currents of the wild-type NaChBac and F108A mutant channels (*n =* 6–7).

Our previous study showed that JZTx-27 bound with the S3–4 extracellular loop of NaChBac, with the 103th phenylalanine (F103) being the key residue [29]. We analyzed the key residues in NaChBac for binding JZTx-14 in the S3–4 loop region by using an alanine scan strategy. The sequence alignment of several bacterial Na_V_s determined this loop in NaChBac to be 103–FAGAQFV–109 (Figure 6A, in NaChBac protein sequence numbering). These mutant channels all expressed large currents in CHO-K1 cells. As shown by the representative traces in Figure 6B–G, 1 µM JZTx-14 almost fully inhibited wild-type NaChBac channel currents; however, its potency to F103A, G105A, Q107A, F108A and V109A mutant channels was attenuated. Notably, 1 µM toxin only weakly inhibited the currents of the F108A mutant channel. The dose–response curves also show that JZTx-14 exhibited attenuated potency to all of the mutant channels, with F108A in NaChBac being the key residue for binding with the toxin (Figure 6H,I). The IC_50_ values were 320.0 ± 38.0 nM for wild-type NaChBac (*n* = 5), 832.6 ± 42.1 nM for F103A (*n* = 7), 653.3 ± 92.1 nM for G105A (*n* = 5), 809.8 ± 87.5 nM for Q107A (*n* = 6), 3472.3 ± 195.7 nM for F108A (*n* = 6), and 1367.3 ± 129.3 nM for V109A (*n* = 5) mutant channel, respectively. We further tested the effect of JZTx-27 on the F108A mutant channel, and the IC_50_ was determined as 116.8 ± 11.0 nM, with only a ~2.5-fold change being observed when compared with wild NaChBac (IC_50_ = 46.7 ± 1.9 nM) (Figure 6J–L, *n* = 6–7). These data suggest that JZTx-14 and JZTx-27 bind to different key residues in NaChBac, although their interacting surfaces in the channel might partially overlap.

## 3. Discussion

This study purified and characterized JZTx-14 from the venom of *C. jinzhao* as a broad spectrum Na_V_s toxin that acted on mammalian Na_V_1.2–1.9 subtypes and the bacterial NaChBac channel. It showed multiple phenotypes in tested Na_V_s, with the toxin inhibiting the fast inactivation of the Na_V_1.9 channel and mainly inhibiting the peak currents of Na_V_1.2–1.8 and NaChBac. For Na_V_1.2–1.7 subtypes, the toxin also slightly inhibited their fast inactivation. The promiscuous mammalian Na_V_s inhibitory activity of JZTx-14 resembled that of GrTx1 and GsAF1 [34]. However, unlike JZTx-14, submicromolar concentration of GrTx1 and GsAF1 were not active on the Na_V_1.5 channel. JZTx-14 acted on mammalian Na_V_s and the NaChBac channel as a gating modifier and the DII S3–4 loops of mammalian Na_V_s and the NaChBac S3–4 loop were involved in toxin association; however, the action mode of JZTx-14 on mammalian Na_V_s was a departure from classical site 4 toxins. We suggest that JZTx-14 might be useful in exploring the pharmacology of Na_V_s as follows.

In this study, JZTx-14 seemed to bind to two sites in mammalian Na_V_ channel to inhibit both the activation and fast inactivation of the Na_V_1.2–1.7, especially for the Na_V_1.2 and Na_V_1.3 channels. This is not surprising, as several toxins such as ProTx-I, ProTx-II, and TsVII were shown to interact with multiple regions in Na_V_s [35,36]. As JZTx-14 mainly inhibited the peak currents of the Na_V_1.2–1.8 channels, its primary and high-affinity binding site in them might be site 1 or site 4 [10]. Guanidinium toxins including TTX and STX, as well as µ-conotoxins including KIIIA, SIIIA, and PIIIA, were two types of site 1 toxins that acted by physically occluding the Na_V_ pore [37,38]. We compared the action modes of JZTx-14 and TTX on the Na_V_1.2 channel in Figure 3. Although JZTx-14 resembled TTX in state-independent binding with Na_V_, and JZTx-14 resembled KIIIA in irreversible binding with Na_V_ [39], the fact that strengthening depolarization reopened the toxin-bound channels strongly supported that JZTx-14 was a gating modifier toxin associating with the Na_V_ DII voltage sensor and trapping it in the deactivated state. Additionally, the Na_V_1.5/1.9DIIS3–4 chimeric channel clearly showed that the DII S3–4 extracellular loop is involved in JZTx-14 association. However, the observations that JZTx-14 irreversibly and state-independently blocked Na_V_s were departures from the action mode of classical Na_V_ site 4 toxins, such as HNTx-III and HWTx-IV [32,33]. As it can be expected that the binding of JZTx-14 to the surface of the Na_V_ DII voltage sensor could be easily washed off by bath solution and easily dissociated by voltage sensor conformation change, both of these were not observed in our experiments. Moreover, the irreversible binding of JZTx-14 to Na_V_1.2–1.8 could not be simply explained by its potential membrane-binding capability, as the action of JZTx-14 on the NaChBac channel was reversible. It is possible that JZTx-14 embedded into the membrane and bound to a deep region in the voltage sensor of Na_V_1.2–1.8 by recognizing a common structure in these channels; however, this binding might be silent, and the toxin inhibitory effect on Na_V_s requires the participation of the S3–4 extracellular loop. We raised a model that JZTx-14 seated in a toxin pocket that was deep within the voltage sensor, and used another surface to interact with and trap the S3–4 loop in its deactivated state from the inside and accordingly impeded the channel activation. This model needs to be validated by more solid data in future studies, and differs from that of the proposed action mode of classical site 4 toxins such as β-scorpion toxin CssIV, in which the toxin binds to the S1–2 and S3–4 loops cleft from the extracellular side [40].

The mutation study showed that JZTx-14 acted on NaChBac by binding to the S3–4 extracellular loop, with the 108th phenylalanine (F108) being the key residue. This action mode resembled the previously reported NaChBac channel toxin JZTx-27, although their key residues in the channel were different [29]. We speculate that JZTx-14 trapped the NaChBac voltage sensor in the deactivated state as it positively shifted the activation kinetics. Indeed, the voltage sensor trapping mechanism is a common mode of gating modifier toxins acting on voltage-gated ion channels [41]. This might be a use-dependent effect of the shifted NaChBac inactivation in Figure 5D, since in a classical two-pulse (condition and test pulses) protocol for assessing channels’ steady-state inactivation, fewer channels were activated in a condition pulse in the JZTx-14 group, however, those toxin-bound channels that were silent in the condition pulse were activated in voltages strong enough to drive them open. The bacterial Na_V_s were inactivated via a C-type inactivation mechanism, in which the collapse of the selectivity filter served as an inactivation gate [42]. The stability of the selectivity filter is thought to affect the C-type inactivation rate of the bacterial sodium channels [43]. The data that JZTx-14 slowed the inactivation of the NaChBac/G105A and NaChBac/F108A mutant channels suggested that the toxin stabilized their selectivity filter; however, the underlying mechanism is currently unknown. 

The bacterial Na_V_s were deemed to be ancestors of mammalian 24TMs voltage-gated calcium and sodium channels [44,45]. Several lines of evidence suggest that their pharmacology might be conserved to some extent. For instance, NaChBac pharmacologically resembled L-type mammalian calcium channels as revealed by two dihydropyridines, nifedipine and nimodipine [14]. Additionally, the local anesthetics lidocaine, QX-314, benzocaine, and ranolazine inhibited the currents of mammalian Na_V_s and NaChBac [46]. Furthermore, the volatile anesthetic isoflurane inhibited NaChBac currents at concentrations that are comparable with those that block mammalian Na_V_s, and it was assumed that the isoflurane binding sites in NaChBac and mammalian Na_V_s are conserved [28]. In this study, the toxin JZTx-14 also inhibited NaChBac and mammalian Na_V_s with similar affinity, but with a distinct mechanism of isoflurane. Whether or not the toxin used the same toxin surface for interacting with these two disparate types of channels remains to be explored.

## 4. Materials and Methods

### 4.1. Toxin Purification and N-Terminal Sequence Determination

The venom of the spider *C. jingzhao* was collected by an electric stimulation method as previously described [47]. The collected venom was lyophilized and stored at −80 °C, and was dissolved in ddH_2_O to a final concentration of 5 mg/mL immediately before being subjected to RP-HPLC purification. The first round of semi-preparative RP-HPLC purification was performed in a Hanbon HPLC system (Hanbon HPLC system equipped with NP7000 serials pump and NU3000 serials UV/VIS detector, Hanbon Sci. & Tech., Huai’an, China) by using a C18 column (10 × 250 mm, 5 μm, Welch Materials Inc., Shanghai, China) and a 45-min linear acetonitrile gradient from 10% to 55% at a flow rate of 3 mL/min. The fraction containing JZTx-14 was collected, lyophilized, and subjected to the second round of RP-HPLC purification in Waters 2795 HPLC system (Waters Corporation, Milford, MA, USA) by using an analytic C18 column (4.6 × 250 mm, 5 μm, Welch Materials Inc., Shanghai, China) and a 24-min linear acetonitrile gradient from 20% to 44% at a flow rate of 1 mL/min. The JZTx-14 store solution was made by dissolving lyophilized toxin in sterile ddH_2_O, and the toxin concentration was determined using the Enhanced BCA Protein Assay Kit following the manufacturer’s instruction (Beyotime Institute of Biotechnology, Shanghai, China). The standard curve of the assay was created using synthetic HWTx-I toxin of known concentration. The N-terminal 11 amino acid sequence of JZTx-14 was determined by Edman degradation in an automatic protein sequencer (Applied Biosystems/PerkinElmer Life Sciences Procise 491-A, PerkinElmer, Inc., Waltham, MA, USA).

### 4.2. Mass Spectrometric Analysis and Toxin C-Terminal Sequence Determination

The purity and molecular weight of the purified JZTx-14 was analyzed in an AB SCIEX TOF/TOF^TM^ 5800 system (Applied Biosystems, Foster City, CA, USA). Briefly, 1 µL JZTx-14 sample solution was mixed with 1 µL saturated CCA (α-cyano-4-hydroxycinnamic acid) solution, pointed onto a sample plate, and then subjected to mass spectrometric analysis in positive reflectron mode. The initial laser intensity was set to 3800, and was finely adjusted to obtain a good resolution and signal-to-noise ratio. Mass calibration was achieved using an external standard. We determined the C-terminal sequence of JZTx-14 by combining chymotrypsin digestion and LC-MS analysis in a Q Exactive^TM^ mass spectrometer (Thermo Fisher, Waltham, MA, USA). Briefly, 10 µg JZTx-14 was reduced, alkylated, and digested with chymotrypsin and subjected to LC-MS analysis. The data was searched against the venom gland cDNA library database of *Chilobrachys jingzhao* by using MaxQuant.

### 4.3. Constructs and Transfection

The cDNA clones of bacterial Na_V_s NaChBac, NsvBa, NaVPz and NaVSp were from professor David E Clapham lab (Janelia Research Campus, Howard Hughes Medical Institute, Ashburn, VA, USA), and were cloned in a pTracer-CMV2 vector. The mammalian Na_V_ cDNA clones (Na_V_1.2–Na_V_1.8) were from professor Theodore Cummins lab (Stark Neurosciences Research Institute, Indiana University School of Medicine, Indianapolis, IN, USA), and were cloned in the pCDNA3.1 or pCMV-blank vectors. The Na_V_1.8/1.7L5 channel and the Na_V_1.9-EGFP channel were as described in our previous studies [30,31]. Mutations were made using the site-directed mutation method. Briefly, the channel plasmid was amplified by PCR using a pair of oppositely-directed primers containing the designed mutation site with KOD Fx (TOYOBO Co., Ltd., Osaka, Japan), then digested with FastDigest DpnI (Thermo Fisher, Waltham, MA, USA) to remove the methylated template. A total of 10 µL digestion mix was used to transform 100 µL *E. coli* DH5α chemical competent cells. All of the mutants were sequenced to ensure that the correct mutations were made. The CHO-K1 cells and HEK293T cells were used for bacterial Na_V_s and mammalian Na_V_s Na_V_1.2–Na_V_1.8 heterologous expression, respectively. Cells were cultured under standard conditions (5% CO_2_, 37 °C) in a humidified incubator and transfection was performed when cells reached 80–90% confluence. All of the transfections were performed using Lipofectamine 2000 following the manufacturer’s instructions (Invitrogen, Carlsbad, CA, USA). Four to six hours after transfection, cells were seeded onto PLL-coated coverslips. Twenty-four hours after transfection, cells were ready for patch-clamp analysis. ND7/23 cells were used for Na_V_1.9-EGFP chimeric channel expression and the conditions were as previously described [31].

### 4.4. Whole-Cell Patch Clamp Recordings

Whole-cell current recordings were performed in an EPC10 USB patch-clamp platform (HEKA Elektronik, Lambrecht, Germany). The recording pipets were prepared from glass capillaries (thickness = 0.225 mm) with a PC-10 puller (NARISHIGE, Tokyo, Japan), and the pipet resistance was controlled to be 1.5–3 MΩ. For recording Na_V_ currents, the bath solution contained (in mM): 140 NaCl, 2 CaCl_2_, 1 MgCl_2_, 5 KCl, 10 glucose and 20 HEPES (pH = 7.3); the pipette solution contained (in mM): 140 CsF, 1 EGTA, 10 NaCl and 10 HEPES (pH = 7.3). TTX was added to bath solution to a final concentration of 1 µM when recording Na_V_1.9 currents. Unless otherwise indicated, all chemicals were products of Sigma-Aldrich (St. Louis, MO, USA). After breaking in, the serial resistance was controlled to be less than 10 MΩ, the voltage error was minimized by using 80% serial resistance compensation, and the speed value for compensation was 10 µs. To minimize the fast capacitance, only the tip of the pipet was filled with pipet solution, and the artificial capacitance effect was canceled by using the computer-controlled circuit of the amplifier. Data were acquired by the PatchMaster software (HEKA Elektronik, Lambrecht, Germany) and analyzed by Sigmaplot 10.0 (Systat Software, Inc., San Jose, CA, USA), Igor Pro 6.10A (WaveMetrics, Inc., Lake Oswego, OR, USA) and Graphpad Prism 5.01 (GraphPad Software, Inc., La Jolla, CA, USA).

### 4.5. Data Analysis

Data were presented as mean ± SEM. N was presented as the number of separate experimental cells. The G–V and SSI curves were fitted by a Boltzmann equation:

y = y_steady_ + (y_(0)_ − y_steady_)/(1 + exp[(V − V_1/2_)/K]) (1)

where V_1/2_, V and K represent the midpoint voltage of kinetics, the test voltage and the slope factor, respectively. The dose–response curves were fitted by a Hill logistic equation to estimate the potency (IC_50_) of the toxin.
